# Older Adults’ Willingness to Adopt Technologies

**DOI:** 10.1093/geroni/igaa057.1824

**Published:** 2020-12-16

**Authors:** Joseph Sharit, Sara Czaja, Jerad Moxley

**Affiliations:** 1 University of Miami, Miami, Florida, United States; 2 Weill Cornell Medicine, New York, New York, United States

## Abstract

Willingness to adopt technology is an important precursor to technology adoption. This talk will present findings from a study which examined 187 older adults’ willingness to adopt a variety of mobile technologies supporting domains such as transportation, health/wellness, and lifelong learning. Participants aged 65 years and older, including 144 females, were presented with Power Point slides describing each of five technologies, and subsequently rated each technology on their willingness to adopt it as well as on the technology’s perceived value, the perceived mental effort required to learn it, confidence in one’s ability to learn it, the degree of help available from family/friends for help learning it, and privacy concerns. Other measures, including self-assessment of skills, technology readiness, technology skills, and cognitive abilities, were also collected. Interrelationships among these and other study variables will be presented as a basis for a model for predicting older adults’ willingness to adopt these technologies.

